# Evaluation of Tracheal Intubation Skill Acquisition in Junior Residents During Anesthesiology Training: An Observational Study

**DOI:** 10.7759/cureus.85204

**Published:** 2025-06-01

**Authors:** Yuko Akanuma

**Affiliations:** 1 Nursing, International University of Health and Welfare, Tokyo, JPN

**Keywords:** advance practice nurse, airway management, medical education, simulation training, task shifting

## Abstract

Background: Anesthesiology training during the residency program is a requirement for junior residents; however, due to only one or two months of anesthesiology training, the experiences of junior residents tend to vary from individual to individual. Furthermore, the decision of skill acquisition level is left to the discretion of a clinical teacher. In most studies, researchers evaluate tracheal intubation based on the number of experiences, success rate, and time required for tracheal intubation. It is difficult to standardize because conditions for tracheal intubation rely on information about a patient’s airway and individual surgical cases. For this reason, it is also difficult to determine clear criteria for tracheal intubation proficiency. We hypothesized that skill acquisition level can be clarified by evaluating tracheal intubation training of junior residents.

Methods: An observational study was conducted on 31 junior residents who participated in the junior residency training in the department of anesthesiology. This research was conducted at our simulation center between November 2021 and March 2023. On the first day of anesthesiology training, participants performed a series of procedures, from tracheal intubation to the connection of artificial respiration, following the orientation for airway management with a simulator. During these procedures, each participant’s movement and laryngoscopy maneuver within the mouth were recorded on video; later, specialist physicians evaluated each performance using an evaluation sheet. On the last day of the two-month training period, participants repeated the same procedures as on the first day of anesthesiology training. Total score based on the evaluation sheet was selected as the primary endpoint, and the time required for tracheal intubation and scores by objectives were selected as the secondary endpoints. Furthermore, questionnaires were administered before and after anesthesiology training. Multiple regression analysis was used to analyze questionnaire results, which determined if there is a causal relationship between variables affecting participants’ satisfaction in anesthesiology training.

Results: The scores were compared between before and after anesthesiology training. When compared to the scores before anesthesiology training, the total score showed a significant increase (8.9±8.5, 95% CI (5.8-12.1), p<0.01), and the score in the time required for tracheal intubation showed a significant decrease after anesthesiology training (58.9±49.7, 95% CI (40.6-77.2), p<0.01). According to the questionnaire results from before and after anesthesiology training, a factor that affected an increase in the score of tracheal intubation skills was the time required for tracheal intubation (adjusted R²=0.1, Akaike information criterion (AIC)=87.5). Factors that affected participants’ satisfaction in anesthesiology training were affiliated department, number of skills experienced, and skills’ satisfaction (adjusted R^2^=0.56, AIC=68.5).

Conclusions: We verified the hypothesis that tracheal intubation skill acquisition following anesthesiology training can be visualized by using the evaluation sheet. In the future, it is necessary to consider management that can experience various skills based on the points of airway management education in the junior residency training. In addition, it is also necessary to establish an education system that can increase interest in anesthesiology training.

## Introduction

Two months of anesthesiology training are mandatory in the residency program for junior residents. On the first day of training, the residents receive airway management orientation using a simulator, followed by tracheal intubation training in surgical anesthesia. Anesthesiology training also has the advantage of providing experience in other advanced technical skills, such as epidural anesthesia and central venous catheter insertion. Anesthesiology training also provides a foundation in safety management, including the prevention of medication errors and non-technical skills with multidisciplinary staff [1,2].

Tracheal intubation, typically among technical skills, is an essential skill not only during surgery but also during emergency care, intensive care, and lifesaving in general wards. Although surgical anesthesia provides daily experience with tracheal intubation, few departments outside of anesthesiology offer educational training. In addition, according to the American Heart Association’s cardiopulmonary resuscitation guidelines, tracheal intubation is an important technique for proper airway clearance and oxygenation during resuscitation, as well as for the prevention of aspiration pneumonia. Proficiency in this requires appropriate initial training and experience [3].

Anesthesiology training is short, only lasting one to two months, and the extent of experience beyond this is likely to vary. The number of times the procedure has been previously performed, the success rate, and the time required for tracheal intubation are used as indicators to evaluate proficiency in this technique. However, since these vary greatly depending on the characteristics of patient airways and specific surgical cases, it is difficult to define a general standard for proficiency in this technique. As a result, clear standards are currently lacking.

Using tracheal intubation skill acquisition as a decision, we referred to the educational guidelines of the Japanese Society of Anesthesiologists (Basic Course in Airway Management) [4] and the assessment tools of the American Council for Accreditation of Medical Education's Anesthesia Education Standards Form (Milestones) [5,6] to create an assessment chart tailored to our hospital environment. This chart was used to conduct a pilot study in 2021. In the present study, we verified the reliability and validity of this assessment chart. On the first and last day of training, tracheal intubation and ventilation training on a simulator using the assessment chart was conducted twice to document technical progress. In addition, an anesthesiology questionnaire was administered before and after training was used to document the number of procedures performed and the residents’ technical scores, as well as to allow residents to evaluate their satisfaction with their training.

The aim of this study was to evaluate skill acquisition in anesthesiology residents during intubation training using a simulator. The hypothesis is that residents’ skills and evaluation scores will be higher after training than before. A questionnaire will be administered pre- and post-training to comprehensively evaluate the causal relationship between the number of times the procedure is performed and skill acquisition, as well as resident satisfaction with the training content. The acquisition of appropriate tracheal intubation skills by residents is key to saving patients’ lives and improving medical safety in the future.

This study was presented as a conference abstract at the American Society of Anesthesiologists Annual Meeting, October 19, 2024.

## Materials and methods

The study participants were comprised of 31 residents undergoing initial training in anesthesiology. The study was conducted at the simulation center of St. Luke's International Hospital, Tokyo, Japan, from November 2021 to March 2023.

Training consisted of a practical exercise using the Laerdal Airway Management Simulator (Laerdal Medical AS, Stavanger, Norway) on the first day, followed by a lecture-style explanation of the technique. The consenting residents were asked to perform a series of actions, from mask ventilation to tracheal intubation and ventilator connection. The training session was recorded from two angles: the residents' actions during the sequence from mask ventilation to tracheal intubation and connection to the ventilator, and the intubation procedure in the oral cavity. The training instruments consisted of a Macintosh laryngoscope with a disposable bronchoscope glued to it and an Ambu® bronchoscope monitor (Ambu A/S, Ballerup, Denmark).

We ensured that the faces of the residents were not captured in the images, taking care to prevent individual identification. The video recordings were subsequently scored by a third-party anesthesiologist using an evaluation chart. On the last day of training, residents were asked to perform the same simulator tracheal intubation procedure as on the first day. The session was recorded and subsequently scored by the same anesthesiologist. The final score was based on the successful completion of each of the five goals in the assessment chart (Table 1).

**Table 1:** Endotracheal intubation skill evaluation chart Each item is scored on a five-point scale, with a total of 20 items for a maximum of 100 points. Decimal scores are also acceptable. Scoring criteria are as follows: One point: unable to perform independently; critical errors such as esophageal intubation (beginner level); Two points: able to perform with physical assistance; Three points: able to perform with verbal guidance (general competency level); Four points: able to perform independently; Five points: performs skillfully (specialist level)

Goal		Score
Goal 1: To be able to assume a position suitable for tracheal intubation		
1	Position the patient's head appropriately (sniffing position, inserting pillows for head and shoulders, etc.)	/5
Goal 2: To be able to properly insert a laryngoscope		
2	Holding the laryngoscope correctly	
3	Cross-finger opening	
4	Insertion from the right corner of the mouth, the tongue is moved to the left and reaches the midsection.	
5	Recognizes the epiglottis valley and can insert the blade	
6	Appropriate application of force to the epiglottis valley	
	Subtotal	/25
Goal 3: Obtain an appropriate airway field of view		
7	Blade position is appropriate, neither deep nor shallow	
8	Blade is in the midsection	
9	Lift the epiglottis without tongue protrusion	
10	The initial laryngeal deployment angle is appropriate (about 45 degrees)	
11	Vocal cords are visible	
12	Number of laryngeal deployments (Note! If you can do it once, you get 5 points)	
	Subtotal	/30
Goal 4: To be able to correctly insert an intubation tube into the trachea and start artificial respiration		
13	No contact with other tissues during tube insertion	
14	Insert the tube to the proper depth	
15	Inject cuff and confirm placement in trachea (chest elevated, etCO2, auscultation, cuff leak)	
16	Initiate manual or mechanical ventilation	
17	Proper skin fixation with tape	
18	Number of times the tube is inserted (Note: If you can do it once, you get 5 points)	
	Subtotal	/30
Goal 5: To avoid trauma during intubation		
19	Not applying excessive force during laryngoscopy or tube insertion	
20	Laryngoscope does not pinch lips or cause dental damage	
	Subtotal	/10
	Overall score	points
	The time required	seconds

The primary endpoint was the overall score based on the assessment chart, while the secondary endpoint was the time required for the procedure.

Statistical analysis to confirm differences between the pre- and post-training scores was performed using a paired t-test. In addition, the overall evaluation and satisfaction scores were analyzed by multivariate analysis. The following is a pre- and post-training questionnaire that was used for the analysis: Please indicate your level of interest in anesthesiology on a numerical scale of one to 10. Please indicate your level of satisfaction with learning the technology on a numerical scale of one to 10. Please indicate your level of overall satisfaction with the anesthesiology training on a numerical scale of one to 10.

Multiple correlations were then calculated between the five goals in the assessment chart and each indicator, as well as the relationships between the pre- and post-training scores. This study was approved by St. Luke's International Hospital's research ethics review committee (approval number: 21-R087).

## Results

In this study, tracheal intubation and ventilatory training were filmed before and after the first and last day of training, and the overall score and time required to complete the procedure were compared based on the goals on the assessment chart. Among the 31 participants, 15 were male and 16 were female, with 15 belonging to the internal medicine unit and 16 to surgery. Twenty were in their first year of initial training, and 11 were in their second year, with the second year being their first rotation in anesthesiology.

**Figure 1:**
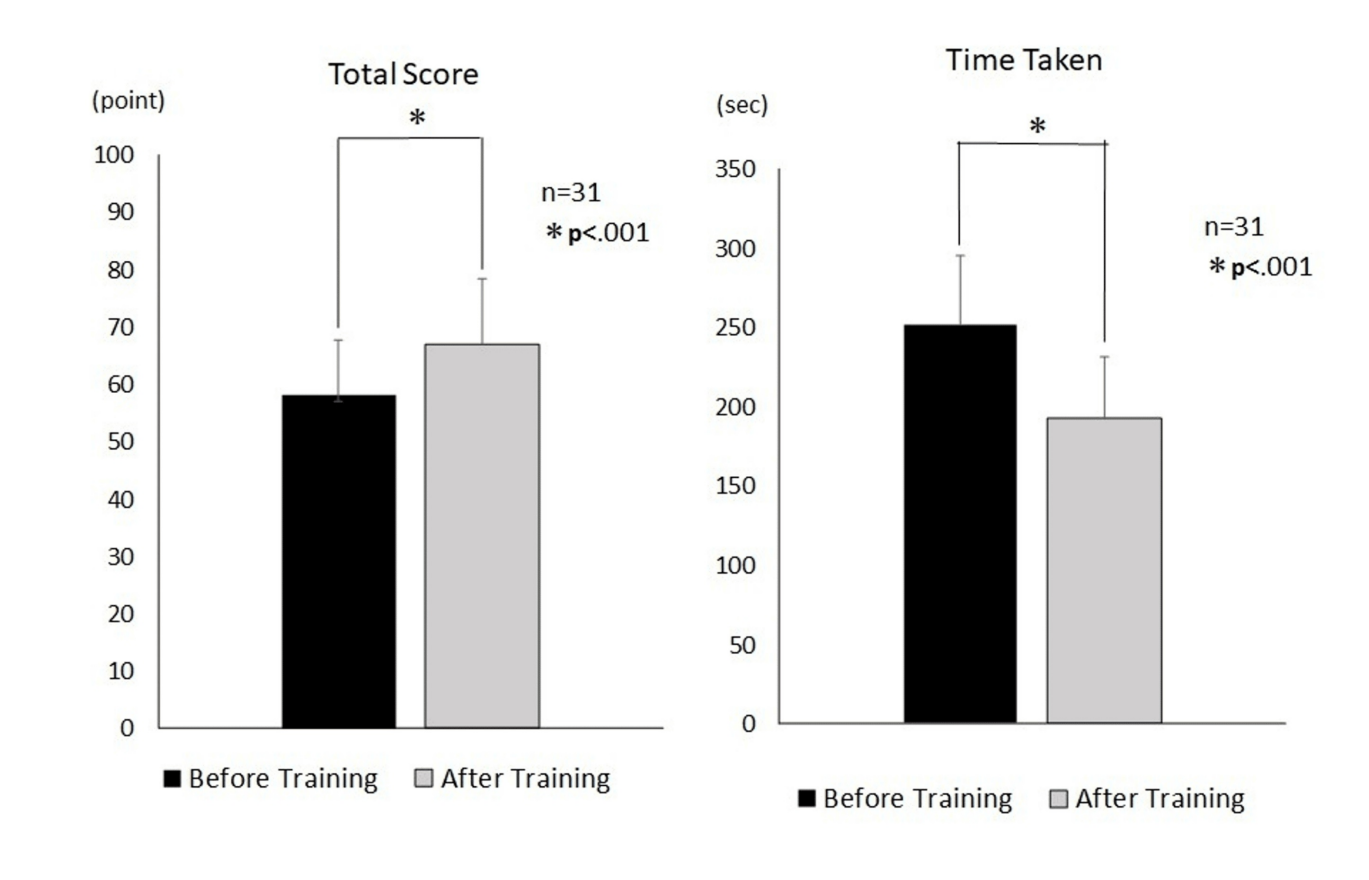
Comparison of the difference in total score and time taken before and after training n=31; paired t-test

The overall score on the primary endpoint assessment chart increased significantly after training (8.9±8.5, 95% CI (5.8-12.1), p<0.01), and the time required from head positioning to intubation and ventilator connection decreased significantly (58.9±49.7, 95% CI (40.6-77.2), p<0.01) (Figure 1).

**Figure 2:**
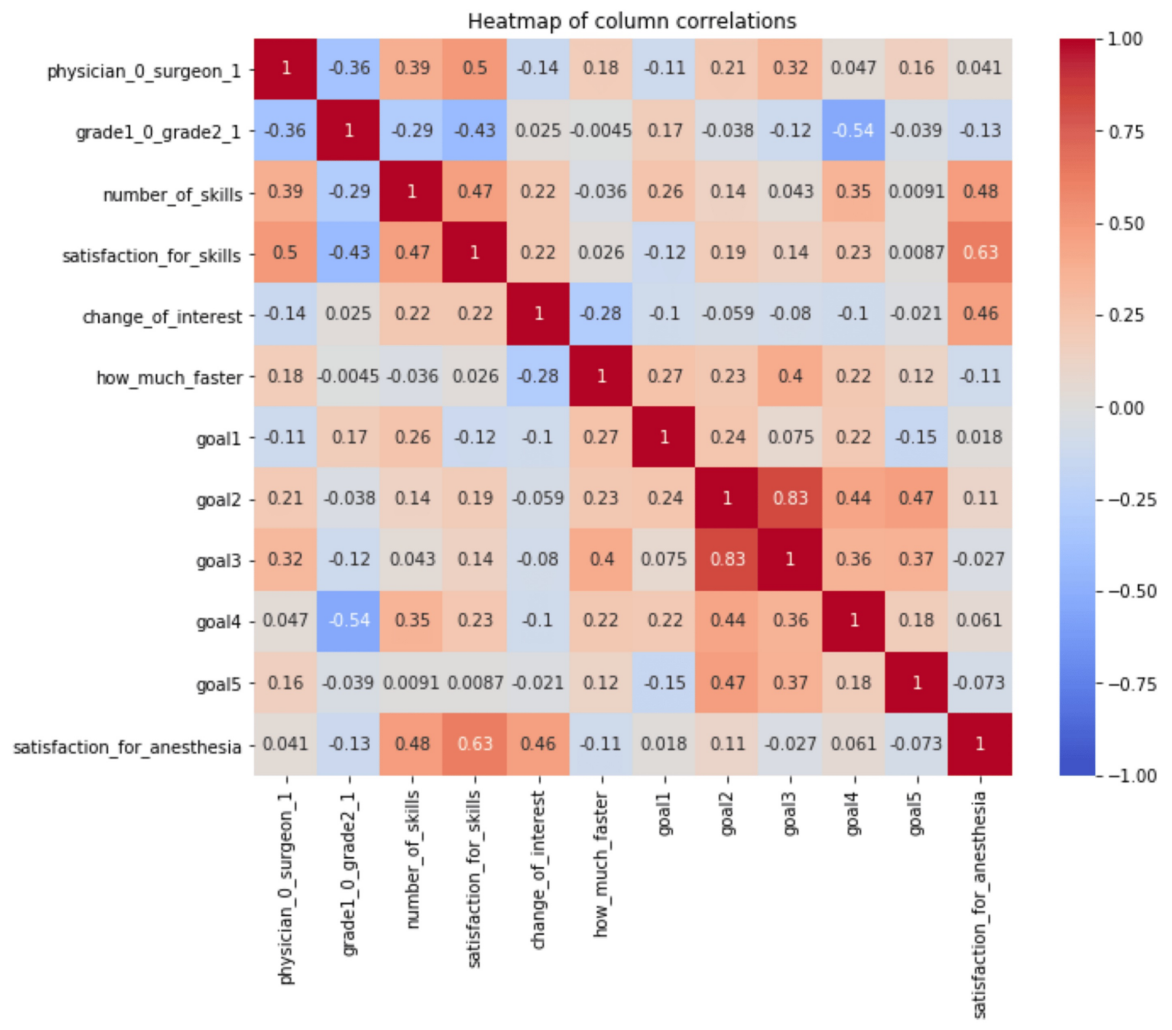
Correlation heatmap between the goals in the evaluation form and each indicator

From these results, the two hypotheses set forth are affirmed. Comparing the scores pre- and post training, these increased significantly for all five goals on the assessment chart (p<0.01). In addition, a heatmap of the correlation between each item showed a strong correlation between Goals 2 and 3 (r=0.83, p<0.05) (Table 2, Figure 2).

**Table 2:** Comparison of pre- and post training evaluations n=31; paired t-test; * p<.01; †p<.001

	Evaluations	Pre-training	Post training	Mean±SE	95%CI
Primary outcome	Total score of Goals 1-5 (points)	58.1	67.0	8.9 ± 8.5 †	5.8 - 12.1
Secondary outcome	Goal 1: Positioning	2.9	3.5	0.6 ± 0.1 †	0.4 -0.9
	Goal 2: Insertion of a laryngoscope	13.0	15.8	2.8 ± 0.5 †	1.7 - 3.7
	Goal 3: Laryngeal view	17.5	19.8	2.3 ± 0.7 *	0.9 - 3,7
	Goal４: Intubation and connect to a ventilator	19.5	21.7	2.1 ± 0.5 †	1.1 - 3.1
	Goal 5: Injury	5.2	6.2	1.1 ± 0.2 †	0.6 - 1.5
	Time taken (seconds)	251.7	192.8	58.9 ± 49.7 †	40.6 - 77.2

**Figure 3:**
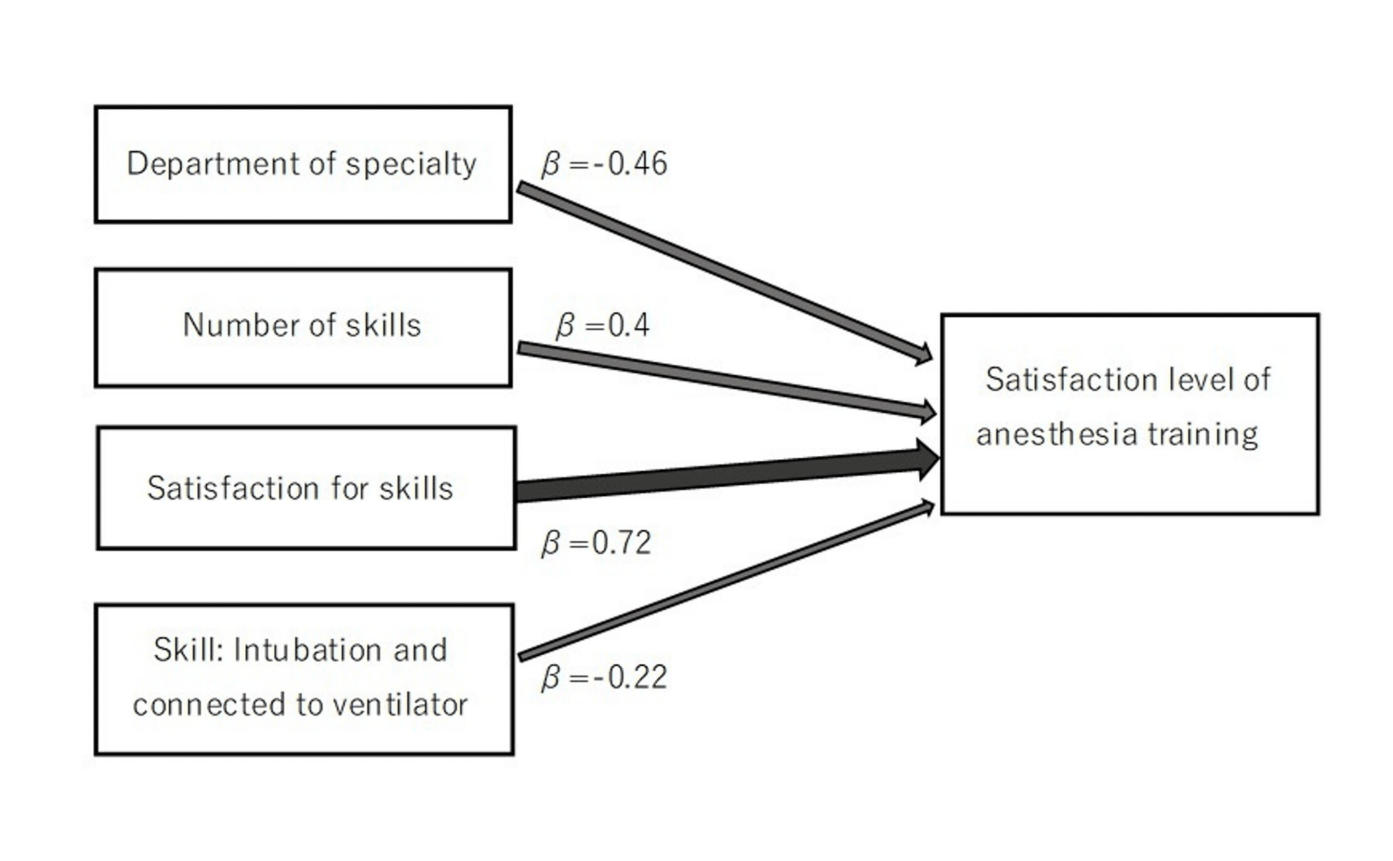
Path diagram: factors that affect the level of satisfaction with anesthesia training β: regression coefficient

Multiple regression analysis was performed on each factor affecting the overall tracheal intubation score and the residents’ satisfaction with the anesthesiology training. The results showed that the only factor affecting the overall score was the time required for the procedure (adjusted R^2^ = 0.1, Akaike information criterion (AIC) = 87.5). The prediction accuracy of the model was judged to be quite low. Factors that influenced training satisfaction included the department in which they were affiliated, the number of times the procedure had been performed previously, satisfaction with the skills experienced, and Goal 4 (adjusted R^2^ = 0.56, AIC = 68.5) (Table 3, Figure 3).

**Table 3:** The factor affecting the satisfaction level of anesthesia training Dependent variable: the satisfaction level of anesthesia training, *p<.05 Goal 4: Intubation and connection to the ventilator skill; AIC: Akaike's information criterion; β: regression coefficient; VIF: variance inflation factor

Parameters	β	SE	p-value	95%CI		VIF
Department of specialty	-0.46	0.19	0.004*	-1.01	-0.21	1.41
Number of skills	0.4	0.08	0.01*	0.05	0.38	1.47
Satisfaction with skills	0.72	0.2	0.001*	0.56	1.39	1.53
Goal 4	-0.22	0.03	0.1	-0.12	0.01	1.17
R	0.78					
R^2^	0.61					
Adjusted R^2^	0.56					
AIC	68.5					

## Discussion

In this study, the acquisition of tracheal intubation skills by residents during their initial hospital training was evaluated using an assessment chart to document their progress and satisfaction. Many hospitals do not currently have any established standardized criteria to determine tracheal intubation skill. As a result, evaluation is typically based solely on the judgment of a specialist. However, this study proposes using an evaluation chart to evaluate and monitor the acquisition of tracheal intubation skills, including an overall score on the assessment chart after training and a record of the time required to complete the procedure. A comparison of the scores before and after training showed that the scores increased significantly across all five assessment criteria (Goals 1-5), suggesting that goal-setting is necessary for skill acquisition. In particular, the results showed a strong correlation between Goals 2 and 3, indicating a close relationship between laryngoscopy and laryngeal deployment.

Previous reports on anesthesiology training have suggested that 57 instances of tracheal intubation are needed to achieve a 90% success rate for this technique [7]. Other studies have also reported that between 43 [8] and 30 cases [9] are needed to achieve an 80% success rate. This is not likely to be achieved in a two-month training program. The number of actual attempts may be more critical than the duration of the training program. Many studies use the number of times the procedure has been performed, the success rate, and the time required to conduct the procedure as criteria for evaluating tracheal intubation skills [10,11].

However, since management in real patients varies depending on the patient's airway status and specific surgical procedures, it would be shortsighted to evaluate success solely based on the ratio of tube insertion and the time required. There is a need to comprehensively evaluate several actions, starting with head positioning of the patient, confirming that the tube is securely inserted, and ending the procedure only after smooth ventilation is confirmed. To this end, we consulted previous protocols studied in similar settings [6,12,13]. Anesthesiology guidelines from around the world may prove to be useful references, but cannot be reduced to specifics. Multidisciplinary procedure evaluation tools, such as Objective Structured Assessment of Technical Skills (OSATS) [14,15] and Direct Observation of Procedural Skills (DOPS) [16-18], score each item on a five-point scale and facilitate nominal and ordinal scale analyses. Current assessment charts analyze up to 20 items using an overall integer score. In the future, the use of larger training cohorts will be essential to solidify the foundations of this assessment while making necessary modifications to develop the best training guidelines.

The results of the training sessions were analyzed using multivariate analysis to identify factors that play important roles in improving the skills and training satisfaction of residents. The time required to complete the procedure was selected as a factor influencing the improvement of the overall score. However, because of the model's low accuracy results, it was not possible to identify factors affecting the overall score. Factors affecting satisfaction with anesthesiology training included the department of junior residents, the number of times the procedure was performed, satisfaction with the skills, and Goal 4. In particular, satisfaction with the skills and the number of times the procedure was performed had a significant impact on satisfaction with the training, suggesting that these two factors play important roles during anesthesia training. Since anesthesiology is the only field in which residents learn advanced skills needed to treat critical patients and respond to emergencies [19], it is clear that the residents had high expectations [20-24].

Japan has been experiencing a significant shortage in physicians since the 2000s due to government healthcare policies, including a shortage in anesthesiologists that continues to this day [25-26]. With day-to-day clinical work taking precedence, the establishment of a specialized training system may still take time. They promoted team medicine, which is now a policy, and collaborated with physicians and nurses while teaching residents and conducting research. Based on this collaborative approach, this study seeks to contribute to the construction of a fulfilling medical education system and the creation of an environment that increases resident satisfaction with anesthesiology training. The purpose of this study was to evaluate skill acquisition during tracheal intubation training conducted by anesthesiology residents.

The limitations of this study include insufficient rigor in the selection and exclusion criteria of participants, inadequate training of evaluators, and a lack of rigor in the research environment. In the future, we aim to establish a standardized environment to ensure reproducibility of techniques and assessments, enabling their use even in patient care sessions.

## Conclusions

This study empirically evaluated the hypothesis that the introduction of a skill assessment chart for simulator tracheal intubation training would increase resident evaluation scores after the training compared to before training, reduce the time required to complete the procedure, and facilitate a visual aid for the degree of skill acquisition.

Questionnaires administered to the residents pre- and post training allowed us to evaluate the causality between the number of times the procedure was performed and the satisfaction with training among the participants. As a future direction, we are considering excluding the average number of attempts required to acquire sufficient intubation skills and using this as a target value for subsequent training sessions. Based on the results of this study, we hope to develop an education system that allows for skill-based learning and an attractive training program for medical residents.

## References

[1] Fayed M, Nowak K, Angappan S, Patel N, Abdulkarim F, Penning DH, Chhina AK (2022). Emergent surgical airway skills: time to re-evaluate the competencies. Cureus.

[2] Krenzischek DA, Clifford TL, Windle PE, Mamaril M (2007). Patient safety: perianesthesia nursing's essential role in safe practice. J Perianesth Nurs.

[3] Schmidt E, Goldhaber-Fiebert SN, Ho LA, McDonald KM (2013). Simulation exercises as a patient safety strategy: a systematic review. Ann Intern Med.

[4] Japanese Society of Anesthesiologists (2024). Educational Guidelines for Anesthesiology, 3rd Edition (Book in Japanese). Educational Guidelines for Anesthesiology, 3rd ed, Chapter(in Japanese).

[5] (2025). Anesthesiology milestones. https://www.acgme.org/Specialties/Milestones/pfcatid/6/Anesthesiology/.

[6] Ryason A, Petrusa ER, Kruger U (2020). Development of an endotracheal intubation formative assessment tool. J Educ Perioper Med.

[7] Konrad C, Schüpfer G, Wietlisbach M, Gerber H (1998). Learning manual skills in anesthesiology: is there a recommended number of cases for anesthetic procedures?. Anesth Analg.

[8] de Oliveira Filho GR (2002). The construction of learning curves for basic skills in anesthetic procedures: an application for the cumulative sum method. Anesth Analg.

[9] Komatsu R, Kasuya Y, Yogo H, Sessler DI, Mascha E, Yang D, Ozaki M (2010). Learning curves for bag-and-mask ventilation and orotracheal intubation: an application of the cumulative sum method. Anesthesiology.

[10] Flynn SG, Park RS, Jena AB (2024). Coaching inexperienced clinicians before a high stakes medical procedure: randomized clinical trial. BMJ.

[11] Yau SY, Chang YC, Wu MY, Liao SC (2021). Does seniority always correlate with simulated intubation performance? Comparing endotracheal intubation performance across medical students, residents, and physicians using a high-fidelity simulator. PLoS One.

[12] Al-Wassia H, Bamehriz M, Atta G, Saltah H, Arab A, Boker A (2022). Effect of training using high-versus low-fidelity simulator mannequins on neonatal intubation skills of pediatric residents: a randomized controlled trial. BMC Med Educ.

[13] Nguyen WT, Remskar M, Zupfer EH, Kaizer AM, Fromer IR, Chugaieva I, Kloesel B (2022). Development and use of an induction of general endotracheal anesthesia checklist assessment for medical students in a clinical setting during their introductory anesthesiology clerkship. J Educ Perioper Med.

[14] (2025). OSATS: The OSATS (objective structured assessment of technical skills) are one of the workplace-based assessment tools (WPBAs) used in O&G training. https://www.rcog.org.uk/careers-and-training/starting-your-og-career/specialty-training/assessment-and-progression-through-training/workplace-based-assessments-wpbas/osats/.

[15] Khaliq T (2013). Reliability of results produced through objectively structured assessment of technical skills (OSATS) for endotracheal intubation (ETI). J Coll Physicians Surg Pak.

[16] Delfino AE, Chandratilake M, Altermatt FR, Echevarria G (2013). Validation and piloting of direct observation of practical skills tool to assess intubation in the Chilean context. Med Teach.

[17] Kamat C, Todakar M, Patil M, Teli A (2022). Changing trends in assessment: effectiveness of Direct Observation of Procedural Skills (DOPS) as an assessment tool in Anaesthesiology postgraduate students. J Anaesthesiol Clin Pharmacol.

[18] Dabir S, Hoseinzadeh M, Mosaffa F (2021). The effect of Repeated Direct Observation of Procedural Skills (R-DOPS) assessment method on the clinical skills of anesthesiology residents. Anesth Pain Med.

[19] Rothkrug A, Mahboobi SK (2025). Simulation Training and Skill Assessment in Anesthesiology. https://www.ncbi.nlm.nih.gov/books/NBK557711/.

[20] Pallansch R, Gaiser RR (2023). A departmentally developed agreement to improve faculty-resident feedback. J Educ Perioper Med.

[21] Huang L, An G, You S, Huang S, Li J (2020). Application of an education model using the WeChat public platform in the standardized training of anesthesiology residents. Ann Palliat Med.

[22] Sun H, Chen D, Warner DO, Zhou Y, Nemergut EC, Macario A, Keegan MT (2021). Anesthesiology residents’ experiences and perspectives of residency training. Anesth Analg.

[23] Scheffel D, Wirkner J, Adler S (2022). Promoting young academics in anesthesiology: factors for an attractive internship (Article in German). Anaesthesist.

[24] Duffy CC, Bass GA, Yi W, Rouhi A, Kaplan LJ, O'Sullivan E (2024). Teaching airway management using virtual reality: a scoping review. Anesth Analg.

[25] Tamai M, Kojima S, Baba Y, Kurahashi K (2024). Variabilities and contentions in anesthesiologists' perspectives on Japanese perianesthesia nurses: a qualitative study. PLoS One.

[26] Akanuma Y (2023). First in Japan: training curriculum for perianesthesia nurses and their clinical practice. J Perianesth Nurs.

